# High Resolution Manometry Guidance During Laparoscopic Fundoplication in Pediatric Surgically “Fragile” Patients: Preliminary Report

**DOI:** 10.3390/children7110215

**Published:** 2020-11-07

**Authors:** Anna Maria Caruso, Mario Milazzo, Vincenzo Tulone, Carlo Acierno, Vincenza Girgenti, Salvatore Amoroso, Denisia Bommarito, Valeria Calcaterra, Gloria Pelizzo

**Affiliations:** 1 Pediatric Surgery Unit, Children’s Hospital, ARNAS Civico-Di Cristina-Benfratelli, 90127 Palermo, Italy; annacaruso81@gmail.com (A.M.C.); mario.milazzo@arnascivico.it (M.M.); vincenzo.tulone@arnascivico.it (V.T.); carlo.acierno@arnascivico.it (C.A.); vincenza.girgenti@arnascivico.it (V.G.); salvatore.amoroso@arnascivico.it (S.A.); denisia.bommarito@arnascivico.it (D.B.); 2 Pediatric and Adolescent Unit, Department of Internal Medicine, University of Pavia, 27100 Pavia, Italy; valeria.calcaterra@unipv.it; 3 Pediatric Unit, “V. Buzzi” Children’s Hospital, University of Milano, 20154 Milano, Italy; 4 Pediatric Surgery Unit, “V. Buzzi” Children’s Hospital, University of Milano, 20154 Milano, Italy; 5 Department of Biomedical and Clinical Science, “L. Sacco”, University of Milano, 20154 Milano, Italy

**Keywords:** high resolution, manometry, pediatric laparoscopic fundoplication

## Abstract

Background: High resolution manometry (HRM), has been recently introduced in clinical practice to detect esophageal intraluminal pressure and esophageal motor function. We evaluated the feasibility and usefulness of intraoperative esophageal HRM during antireflux laparoscopic procedures in pediatric cases with neurological impairment (NI) or esophageal atresia (EA). Methods: From January to November 2019, seven children (5 NI, 2 EA) with gastroesophageal reflux (GER) were enrolled. Data on intraoperative pressure changes of the esophagogastric junction (EGJ) and postoperative follow-up data were collected. Results: Average preoperative LES pressures were not significantly different from postoperative pressures. A sliding hernia was detected in all patients as evidenced by EGJ double peak pressures. Hernia correction after esophageal traction was complete in 71.4% of the patients, and residual hernia (<2 cm) was detected in 28.6%. Postoperative EGJ pressures were higher compared to preoperative sphincteric pressures (*p *< 0.001); in NI patients, higher postoperative values were noted compared to EA (*p* = 0.05). No sliding hernia and/or GER relapses were recorded. Two patients reported dysphagia postoperatively. Conclusions: Intraoperative HRM may optimize esophageal pressure changes during laparoscopic fundoplication. Further studies are needed to confirm the usefulness of a tailored surgical approach to reduce postoperative complications.

## 1. Introduction 

Gastroesophageal reflux disease (GERD) is the most frequent short- and long-term gastrointestinal complication encountered in children and adolescents with severe neurological impairment and/or esophageal atresia repair (EA). The antireflux procedure in these patients can be difficult with a high risk of failure, recurrence or complications [1,2,3,4]. Usually, the fundoplication wrap in patients with persistent or recurring GERD-like symptoms is assessed by endoscopy. The diagnostic accuracy of endoscopy to assess the complications, such as esophageal dysmotility or obstruction of the distal esophagus, or fundoplication relapse is limited. Moreover, radiologic examination is required for the evaluation of bolus passage [5]. Radiologic imaging allows a morphological and functional evaluation of the gastroesophageal junction and does not provide the realistic position of fundoplication wrap or features of the pillars and abnormalities of surrounding structures.

Solid-state high-resolution manometry (HRM), with a series of closely spaced pressure sensors, [6,7,8,9,10,11,12,13], has been recently introduced in clinical practice to detect esophageal intraluminal pressures at different esophageal sites including the esophagogastric junction (EGJ). HRM also helps the surgeon evaluate esophageal motor function in pediatric congenital and acquired esophageal motility disorders [14,15,16,17,18]. HRM accurately evaluates both components of the EGJ (lower esophageal sphincter—LES and crural diaphragmatic sphincter CD). The anatomical relationship and diagnosis of sliding hernia if these components are spatially separated is also feasible [19,20,21]. To date, esophageal intraoperative manometry has usually been used to monitor the high-pressure esophageal zone during achalasia surgery, in both adults and children [22,23].

In this preliminary study, we evaluated the feasibility and usefulness of intraoperative esophageal HRM assessment during each surgical step of laparoscopic antireflux procedures with the aim of monitoring esophageal pressure variation in real time. We also strove to provide technical performance information on HRM, as a new innovative approach, for sliding hernia correction considering the alignment between LES and pillars and for a more tailored antireflux surgical approach in children affected with congenital or acquired esophageal motility disorders.

## 2. Materials and Methods

### 2.1. Patients

We enrolled pediatric surgically fragile patients (≤18 years) with neurological impairment (NI) and children treated at birth for EA and referred to our Pediatric Surgery Units with a diagnosis of severe GERD and indication for surgical treatment. Clinical data for all of the enrolled patients were collected from January to November 2019.

Before surgery, each patient underwent pH Multichannel Intraluminal Impedance to assess GERD and motility [24] and endoscopy for diagnosis of esophagitis and radiology exams for sliding hernia detection. Gastric volume and emptying were also preoperatively obtained. In the postoperative period, all of the patients were submitted to a radiological and clinical evaluation for dysphagia [25].

The study protocol was approved by the Institutional Review Boards (protocol number 354). The study was conducted in accordance with the 1975 Helsinki Declaration, as revised in 2008. All participants and/or their responsible guardians gave written consent after being informed about the nature of the study.

### 2.2. Clinical Data

Clinical data included age, gender, weight, height and body mass index (BMI). Weight and height were measured as previously described in children with NI [26] and with EA [27]. BMI was calculated by dividing the patient’s weight in kilograms by the square of the height in meters.

### 2.3. Anti-Reflux Surgery

Antireflux surgery was indicated in case of severe reflux symptoms (poor growth, digestive or respiratory) and/or esophagitis “non responder” after at least six months of medical therapy with proton pump inhibitors (PPI). 

In NI patients, we decided to perform the Nissen procedure with a complete wrap, while in EA patients, an anterior wrap was performed, based on the presence of esophageal dismotility as evaluated preoperatively with impedance or HRM. When absent, this was an indication to perform a gastrostomy together with the antireflux surgery. The laparoscopic procedure was performed with five trocars and maintenance of pneumoperitoneum pressure values between 8–10 mmHg, utilizing AirSeal^®^.

### 2.4. Intraoperative Manometric Procedure

The HRM study was performed with the ManoScan^®^ ESO High Resolution Manometry (Medtronic-Given Imaging Inc., 25 Hampshire Street, Mansfield, MA, USA) instrument, which is equipped with an imaging catheter consisting of 36 pressure channels. Data acquisition, display and analyses were performed with the manoView Software (Medtronic-Given Imaging Inc., 25 Hampshire Street, Mansfield, MA, USA).

HRM was used to calibrate the entire surgical procedure while the patient was under general anesthesia. The recording time started at intubation and the manometric analysis was simultaneously assessed on the HRM screen, which was placed next to the laparoscopic monitor in such a way that each surgical step (basal evaluation, before and after pneumoperitnoneum, rectraction, mobilization, fundoplication) could be manometrically evaluated. 

The manometric endpoints included LES peak (measured in cm), LES pressure (median value and range), length of LES (cm), localization of CD (cm), identification of sliding hernia, measurement of hernia (measured in cm) (differences between LES and CD localization), postoperative LES position (an effect of traction), EGJ pressure at the end of the procedure, and measurement of residual hernia (if present).

HRM may provide records of the pressures of the esophageal lumen during all surgical steps of the Nissen laparoscopic fundoplication. All esophageal points of the esophagus corresponding to functional abnormalities can be intraoperatively detected by surgeon.

### 2.5. Dysphagia Score

Dysphagia was clinically scored according to the PEDI EAT 10 symptom scale and defined with a score of 4 or higher [25].

### 2.6. Statistical Analysis

Categorical variables were described as counts and percentages. Quantitative variables were expressed as the mean value and standard deviation. The statistical significance of the continuous variable comparisons was assessed using the unpaired Student’s t-test. The comparison of categorical variables was conducted using the chi square test or Fisher’s Exact test if there was a small expected counts (<5). All tests were two-sided, and a *p*-value below 0.05 was considered statistically significant. The data analysis was performed with the STATA statistical package (release 16.1, Stata Corporation, College Station, TX, USA).

## 3. Results

We enrolled seven patients (3 females and 4 males, mean age 10.0 ± 4.4), five with NI and two with EA. The features of the subjects are reported in Table 1. 

### 3.1. Preoperative Data 

As reported in Table 1, a gastrostomy tube was already present before antireflux surgery in four patients (three patients were exclusively fed enterally); one NI patient, who was not exclusively fed enterally, had a normal score of 4; in one NI patient, a gastrostomy tube with the indication for antireflux surgery was recorded (preoperative score during oral feeding was 10).

None of the EA patients had a gastrostomy tube and all of these patients were orally fed. One patient with EA had severe dysphagia before surgery (score 13); this was confirmed with impedance analysis and preoperative manometry. All of the NI patients had severe scoliosis and none had a preoperative diagnosis of sliding hernia based on radiology/endoscopy exams. In one subject with a suspected hernia, a short esophagus was diagnosed before surgery. 

### 3.2. Surgical Data

As reported in Table 2, one NI patient underwent an antireflux redo surgery for recurrence (previous surgery with a complete wrap four years earlier, bearer of PEG-J). In all of the EA patients and in one NI patient (redo case), we performed an anterior fundoplication, while in all of the other cases a complete fundoplication was carried out.

The HRM intraoperative results are reported in Table 2. The average LES pressures were not modified by pneumoperitoneum.

A sliding hernia was detected in each patient, as evidenced by EGJ double peak pressures. In 5/7 (71.4%) patients, the hernia correction after esophageal traction was complete, with evidence of one high pressure zone at the end of the surgical procedure (diaphragm closure and wrap). In 2/7 (28.6%) patients, the residual hernia was less than 2 cm (1 cm in both cases). The postoperative EGJ pressure values were higher compared to preoperative sphincteric LES pressures (81.4 ± 13.4 vs. 40.2 ± 8.8 cm, *p* < 0.001). In NI patients, higher postoperative values were noted compared to EA (87.2 ± 10.8 vs. 66.5 ± 2.1 cm, *p* = 0.05).

### 3.3. Postoperative Outcome 

No intraoperative complications occurred. All patients completed their follow up at six months with clinical evaluation and radiological assessment. Two patients with EA reported dysphagia in the early postoperative period (respective scores of 19 and 12). This condition was prolonged for two months in one of the two patients (score 9 at two months); however, it resolved spontaneously without further treatment (score of 4 at six months). 

The NI patients with a pathological preoperative score, reported a normal value of 3 after gastrostomy positioning and initiating enteral feeding. None of the patients experienced sliding hernia or gastroesophageal reflux recurrences, nor were any obstructive symptoms or vomiting episodes recorded.

## 4. Discussion

Laparoscopic antireflux surgery in patients with severe GERD resistant to medical therapy is the treatment of choice. Surgical treatment for reflux symptoms may not always correlate with postoperative success in NI children and young patients with comorbidities. Moreover, in “fragile” patients, the surgical team must consider the postoperative complications, which include bloat syndrome, hiatal hernia relapse, and the need to adapt the fundoplication for each patient. The manometric assessment is usually used to detect the esophageal motility disorder, such as esophageal achalasia [28]. This technique has also been proposed as a guide to optimizing the surgical results of antireflux surgical procedure [29,30,31]. 

Conventional manometry systems do not allow for a view of pressures generated by the entire esophagus including its sphincter. With HRM, which does not necessitate the pull through maneuver of the catheter, we get a complete spatial and temporal view of esophageal motor function, by converting the pressure data into a color topographical map. A pressure profile of the entire esophagus and sphincters can be simultaneously viewed in real time from the pharynx to the stomach with UES; while EGJ easily identifies zones of high pressure that are depicted as horizontal bands of warmer color [6,7,8,9,10,11,12,13]. Even though no reference values with HRM are available in pediatric “fragile” patients, the experience in the adults [32] may support the role of this approach to optimize the antireflux surgery outcome in children [29,30,31].

EGJ competence depends on the integrity and interaction of several components: intrinsic LES, extrinsic compression of the LES by the CD, compliance of the EGJ, integrity of the phrenoesophageal ligament and maintenance of an acute angle of His promoting a “flap valve” function. Indeed, separation of the LES from the crura, as observed in hiatus hernia, can reduce efficacy on the antireflux barrier. The EGJ evaluation and sliding hernia diagnosis is classically made by barium swallow esophagogram and/or during an esophagogastrodyodenonoscopy (EGD), but these methods are limited by subjective and indirect evaluation of LES and CD location: endoscopy usually tends to exaggerate the size of a hernia while radiology could underestimate small hernias [33]. During HRM, the EGJ pressure profile is visualized as abrupt pressure transitions. Importantly, the relative localization of the two contractile elements of the EGJ (LES and CD) can be distinguished. Based on observations in control subjects and GERD patients, Pandolfino et al. described three EGJ subtypes [19]. In type 1, there is a complete overlap of the CD and LES with a single pressure peak evident on the plot; in type II, there is a minimal discernible LES-CD separation making a double-peaked spatial pressure variation plot representative of an intermediate condition in which LES-CD separation is >1 cm and <2 cm; in type III, the LES-CD separation is >2 cm, which is the signature of hiatus hernia. HRM has a high sensitivity and specificity (94% and 91%, respectively) for sliding hernia detection also in vivo during open surgery. However, despite this feature, to date, esophageal manometry has only been used for the placement of a pH probe or to rule out an esophageal motor disease prior to antireflux surgery [34,35,36,37]. 

In this preliminary study in children with high risk of complications, we firstly exploited and verified the advantages of HRM during antireflux laparoscopic surgery, in order to accordingly calibrate the dynamic effect of every surgical step during the antireflux procedure. The pressure morphology of the esophagus (length, LES position, basal EGJ with sliding hernia if present), any EGJ pressure changes during surgery and the complete or incomplete correction of the sliding hernia were considered. The intraoperative HRM allows the alignment between LES and pillars; on the contrary, the laparoscopic procedure only guides the vision of esophageal final positioning during Nissen fundoplication.

We showed that the effects on sphincteric pressures may vary according to pathological clinical condition, with higher values in NI compared to EA. Our findings showed that during surgery, EGJ pressures increase by almost 40%. These data suggest that a tailored surgical plan may be necessary in fragile patients. 

HRM was also shown to have a greater sensitivity than radiology or endoscopy in diagnosing esophageal hernia. It was possible for the surgeon to verify the hernia repair by measuring traction of the esophagus during the intervention. LES and CD positioning may be identified by one pressure peak. Complete hernia repair and measurement of residual hernia, if the correction could not be completed, were also feasible. HRM allowed the surgical team to monitor EGJ pressure changes due to diaphragmatic pillar closure and packaging of the wrap. 

The average preoperative length of the CD pillars was found to be less than the LES length, and this was considered evidence of hernia; whereas postoperative intra-abdominal positioning of the LES is correspondence with the CD pillar position was considered evidence of complete hernia correction.

In our patients, high final EGJ pressures were observed, whereas previous reports described a variable period of adjustment after surgery and under conventional manometry [38,39]. We are aware that EGJ pressure results cannot be used as reference values and probably represent a pressure range useful to avoid obstructive complications. 

None of our patients experienced complications after procedures. No control group is included in this preliminary study. However, in the literature, the prevalence of post-fundoplication symptoms, usually due to hiatal tightness distal and esophageal stenosis and slipping wrap above the diaphragm, has been reported in 2–10% of the subjects [40,41,42]. These data support that intraoperative HRM laparoscopic fundoplication may limit postoperative complications and optimize the outcome. Postoperative complications such as crural stenosis, stenosis of the wrap, and intrathoracic wrap migration could be avoided [43].

This preliminary study has several limitations starting with the small number of subjects and two different patient populations. This may have limited the statistical significance, and further studies are mandatory to support the results. Secondly, the absence of normative data on the HRM pressure in pediatrics may limit the interpretation of the results. In addition, despite data from the literature in adults showing that general anesthesia does not interfere on the HRM interpretation [44], in our cases, this aspect has not been considered, and an interpretation could not be excluded. Finally, despite a dynamic follow-up, HRM evaluation would also be useful to calculate IRP; this should include any pathological process, mechanical or functional, that prevents flow across the EGJ (such as a tight wrap after antireflux surgery), which in turn may generate an elevated IRP [45].

## 5. Conclusions

The main advantage of HRM is the ability to guide the traction of the esophagus into the abdomen in order to completely correct the hernia. Esophageal pressure changes during fundoplication are also easily recorded; these data may be used to avoid high residual pressures. Calibration and individualization of the technique, based on intraoperative manometric findings, may further reduce rates of postoperative failure or dysphagia [34]. Adaptative and tailored surgical plans are mandatory and provide information for the surgeon on both the physiology of the esophagus and anatomic conditions, especially in fragile children. Future studies will further determine the clinical implications of this new tool and help surgeons to achieve better functional and clinical outcomes in fragile patients. 

## Figures and Tables

**Table 1:** Clinical features of the enrolled patients prior to antireflux surgery.

Patient	Pathology	Sex	Age (years)	BMI (kg/m^2^)	Type of Nutrition	Dysphagia		Preoperative Gastrostomy Tube
						Pre-Operative	Post-Operative	
1	NI	M	14	15.7	Oral/EN	score < 4	score < 4	Yes
2	NI	F	12	15.3	Oral	score 10	score < 4	No
3	EA	M	6	14.1	Oral	score 13	score 19	No
4	NI	M	10	11.2	EN	nd	nd	Yes
5	EA	F	2	15.8	Oral	score <4	score 12	No
6	NI	M	13	14.5	EN	nd	nd	Yes
7	NI	F	13	17.6	EN	nd	nd	Yes

NI: neurological impairment; EA: esophageal atresia; EN: enteral nutrition; nd: not detected.

**Table 2:** Surgical and intraoperative manometric data.

Patient	Pathology	Surgery	Pre-Operative LES Peak (cm)	Post-Operative LES Peak (cm)	CD Pillars (cm)	Mean Pre-Operative LES Pressure (mmHg)	Mean Post-Operative EGJ Complex Pressure (mmHg)	Increase in Post-Operative EGJ Pressures (mmHg)
1	NI	Nissen	27	31	30–31.5 (30.7)	42 (27–62)	81	39
2	NI	Nissen	31	33.5	34.5–36 (35.7)	33 (29–73)	95	62
3	EA	Anterior	23	29	27–29 (28.2)	38(28–65)	65	27
4	NI(redo)	Anterior	20	25	26–28 (27)	48 (15–55)	102	54
5	EA	Anterior	22	24	24–25.5 (24.7)	29.7 (22.3–37)	68	38.3
6	NI	Nissen	31	35	35–36.5 (35.8)	55 (32–64)	82	27
7	NI	Nissen	35	39	39–40 (39.5)	36 (30–40)	77	41

NI: neurological impairment; EA: esophageal atresia; LES: lower esophageal sphincter; EGJ: esophageal gastric junction; CD: crural diaphragmatic.
